# Supplementary material for: Wbp2 is required for normal glutamatergic synapses in the cochlea and is crucial for hearing
Source: EMBO Mol Med. 2016 Feb 8;8(3):191–207. doi: 10.15252/emmm.201505523 (PMC4772953; doi:10.15252/emmm.201505523)
Supplement: Supplementary file 1 — Expanded View Figures PDF [file EMMM-8-191-s001.pdf]

Expanded View Figures

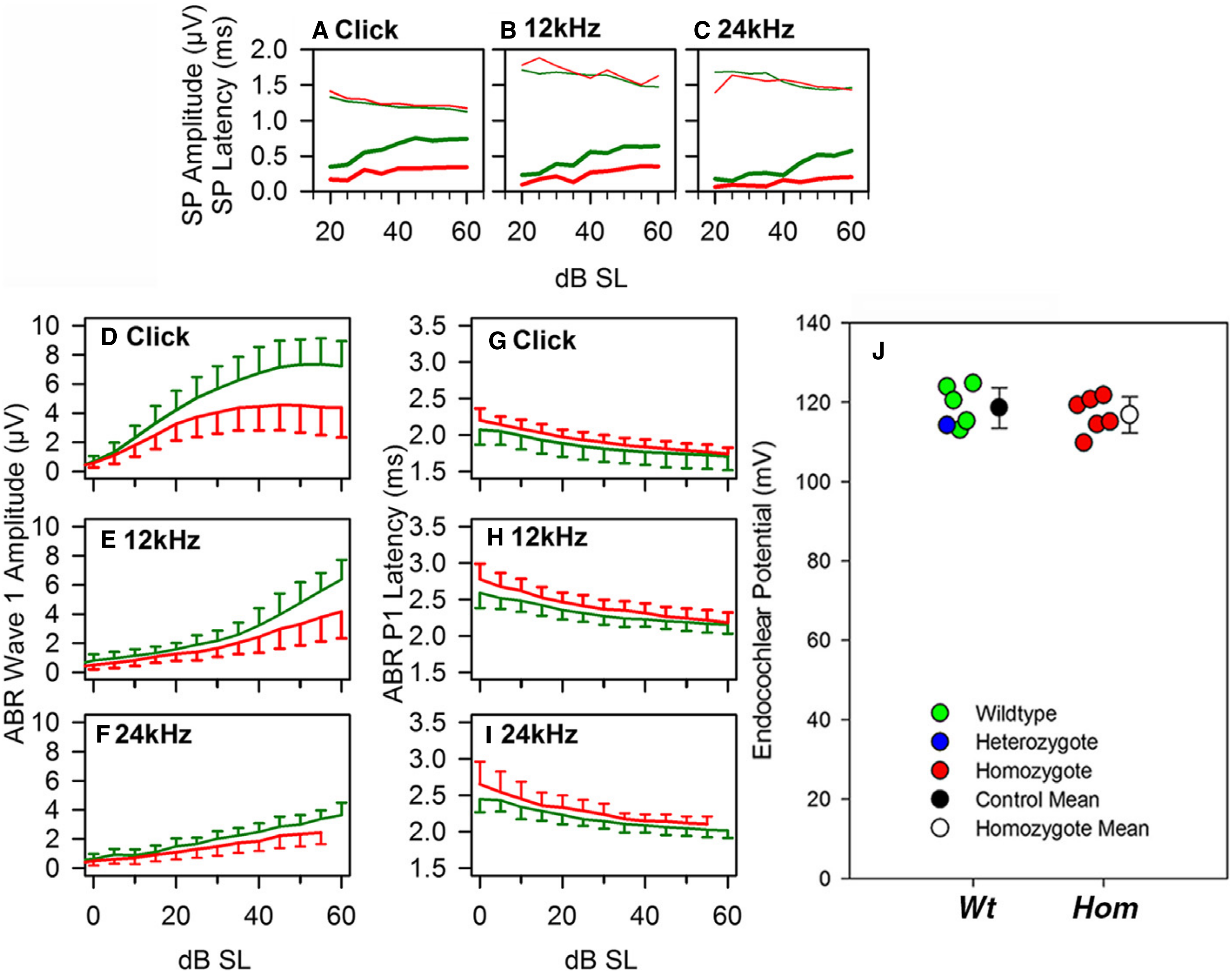

Figure EV1. Supplementary physiology.

A–C SP amplitude (thick lines) and latency (thin lines) are plotted as a function of dB SL, for wt (green, n = 23) and mutant (red, n = 34) responses to clicks (A), 12-kHz tones (B) and 24-kHz tones (C). Values were pooled across stimulus level for statistical analysis. For each stimulus, SP amplitude was significantly reduced in mutants. Click: mean wt SP = 0.61 µV, mean mutant SP = 0.28 µV, t-test P = 0.0000323. 12 kHz: mean wt SP = 0.47 µV, mean mutant SP = 0.25 µV, t-test P = 0.00257. 24 kHz: median wt SP = 0.26 µV, median mutant SP = 0.13 µV, Mann–Whitney rank-sum test U = 6.000 P = 0.003. For each stimulus, SP latency was not changed. Click: mean wt = 1.21 ms, mean mutant = 1.26 ms, t-test P = 0.208. 12 kHz: mean wt = 1.61 ms, mean mutant = 1.68 ms, t-test P = 0.154. 24 kHz; median wt = 1.55 ms, median mutant = 1.54 ms, Mann–Whitney rank-sum test U = 30.500 P = 0.401.

D–F Mean ABR wave 1 amplitude (± SD) is plotted as a function for dB SL for wt (green, n = 23) and mutant (red, n = 34) responses to clicks (D), 12-kHz tones (E) and 24-kHz tones (F). For each stimulus, W1 amplitude was reduced in mutants; Kruskal–Wallis one-way ANOVA on ranks; click, H = 591.579, $P < 0.001$; 12 kHz, H = 631.535, $P < 0.001$; 24 kHz, H = 524.426, $P < 0.001$.

G–I Mean ABR wave 1 latency (±SD) is plotted as a function for dB SL for wt (green, n = 23) and mutant (red, n = 34) responses to clicks (G), 12-kHz tones (H) and 24-kHz tones (I). For each stimulus, positive peak P1 latency was increased in mutants; Kruskal–Wallis one-way ANOVA on ranks; click, H = 606.088, $P < 0.001$; 12 kHz, H = 612.422, $P < 0.001$; 24 kHz, H = 497.363, $P < 0.001$.

J Endocochlear potential was recorded in Hom and Wt, and no significant difference in the values was observed (t-test, wt controls: 119.5 ± 5.2 mV, n = 5, range 113.0–124.7; mutants: 116.8 ± 4.6 mV, n = 6, range 109.7–121.7). P = 0.39526.

© 2016 The Authors

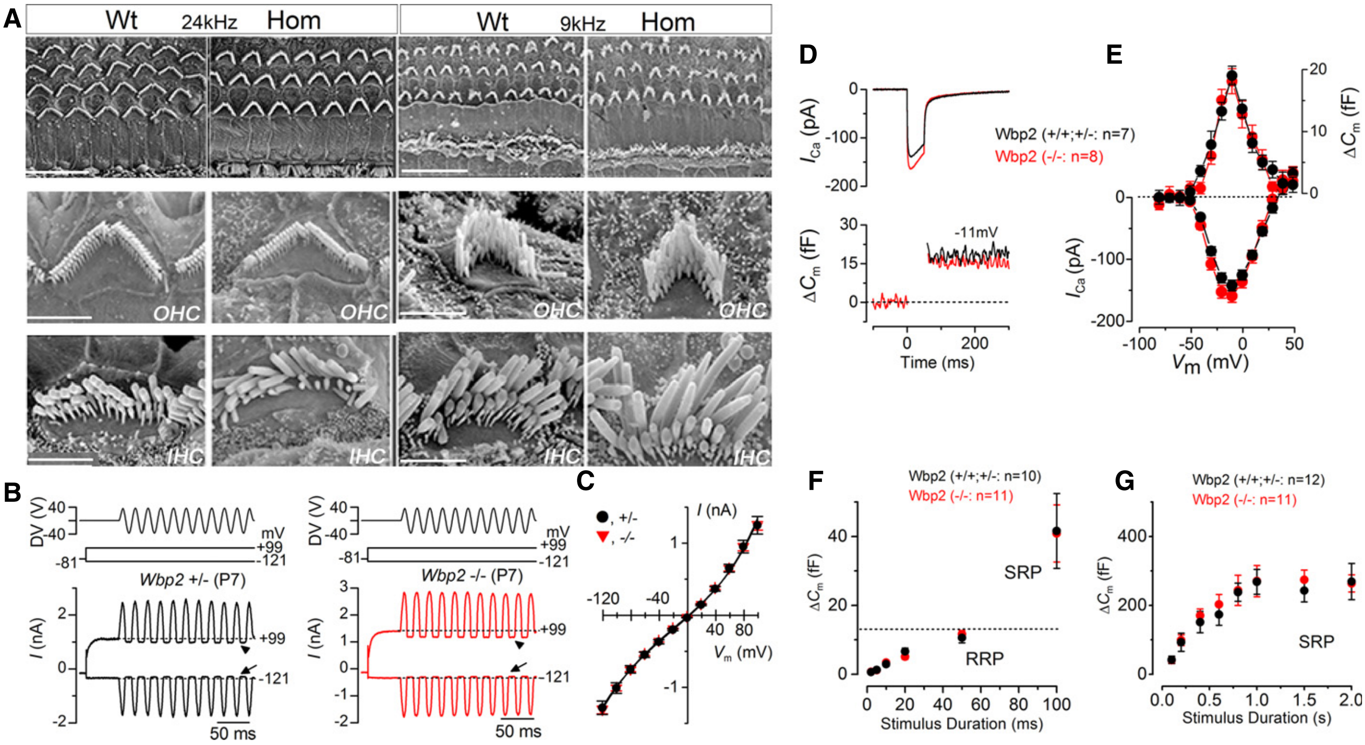

Figure EV2. Normal appearance of hair cell bundles and normal pre-synaptic function in Wbp2-deficient mice.

A SEM showing normal appearance of IHC and OHC hair bundles in 6-week-old Wbp2-deficient mice compared to wt controls, illustrated for the 24-kHz (40% of the length of the cochlear duct from the base) and 9-kHz (80% position) best frequency regions. There was no sign of excess degeneration up to 30 weeks in mutants compared to littermate controls. Scale bars: upper row, 20 μm; middle row, 3 μm; bottom row, 1 μm.

B Saturating mechanoelectrical transducer (MET) currents recorded from a P7 control and a *Wbp2*-mutant IHC by applying voltage steps from −121 mV to +99 mV in 20-mV increments (holding potential −81 mV). For clarity, only two voltage steps are shown. During the voltage steps, hair bundles were displaced by applying 50-Hz sinusoidal force stimuli (the driver voltage, DV, to the fluid jet is shown above the traces). Negative deflections of the DV are inhibitory. The arrows indicate the closure of the transducer channels, that is disappearance of the resting current, during inhibitory bundle displacements. Dashed lines indicate the holding current, which is the current at the holding potential of −81 mV.

C Peak-to-peak current–voltage curves obtained from 11 controls and 5 *Wbp2*-mutant IHCs (P7–P8).

D I_{Ca} and ΔC_m responses from adult (P19–P33) control and *Wbp2*-mutant IHCs from high-frequency region. Recordings were obtained in response to 50-ms voltage steps, in 10-mV increments, from −81 mV. For clarity, only average maximal responses are shown (control: $n = 7$; *Wbp2* mutant: $n = 8$).

E Average peak I_{Ca}-voltage (left axis) and ΔC_m-voltage (right axis) curves from control and *Wbp2*-mutant IHCs (control: $n = 7$; *Wbp2* mutant: $n = 8$).

F Average ΔC_m from 10 control and 11 *Wbp2*-mutant IHCs in response to voltage steps from 2 ms to 100 ms (to around −11 mV) showing mainly the RRP and the initial recruitment of the SRP for the 100-ms step.

G Average ΔC_m from 12 control and 11 *Wbp2*-mutant IHCs in response to voltage steps from 100 ms to 2 s (to around −11 mV) showing the SRP.

Data information: Data are shown as mean ± SD.

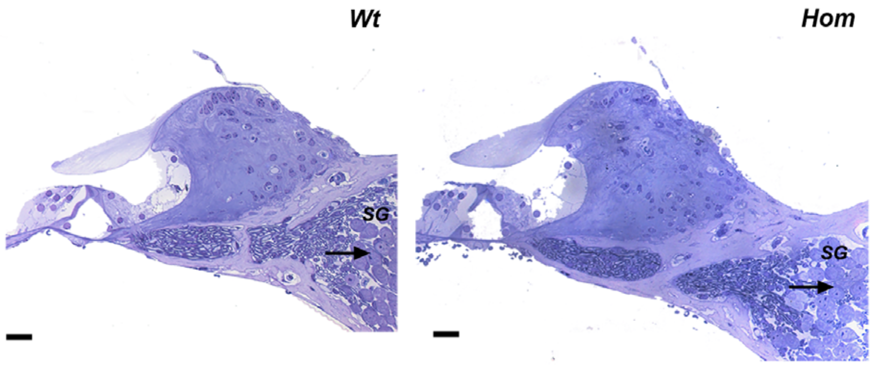

Figure EV3. Histology of organ of Corti.
Semi-thin sections stained with toluidine blue show no cochlear abnormalities and no obvious loss of spiral ganglion cells in Wbp2-deficient mice compared to controls at 4 weeks. Scale bar: 20 μm. SG: spiral ganglion cells.
