# Supplementary material for: Wbp2 is required for normal glutamatergic synapses in the cochlea and is crucial for hearing
Source: EMBO Mol Med. 2016 Feb 8;8(3):191–207. doi: 10.15252/emmm.201505523 (PMC4772953; doi:10.15252/emmm.201505523)
Supplement: Supplementary file 2 — Table EV1 [file EMMM-8-191-s002.docx]

**Table EV1.**

|  | Controls | mutants |
| --- | --- | --- |
| Membrane capacitance (pF) | 8.1 ± 0.8 (6) | 7.3 ± 0.4 (9) |
| Resting potential (mV) | -68.4 ± 1.5 (5) | -66.7 ± 1.4 (9) |
| *I*_K_ at 0 mV (nA) | 9.3 ± 1.2 (5) | 10.7 ± 1.2 (8) |
| *I*_K,f_ at -25 mV (nA) | 1.9 ± 0.3 (6) | 1.2 ± 0.3 (8) |
| *I*_K,n_ at -124 mV (pA) | 211 ± 71 (5) | 158 ± 21 (6) |

**Properties of basal-coil IHCs from P25-P33 Wbp2 mice**. Values are means ± s.e.m.; number of hair cells is in parentheses. *I*_K_ = Delayed rectifier K^+^ current; *I*_K,n_ = Negatively activated K^+^ current carried by KCNQ4 channels; *I*_K,f_ = Ca^2+^-activated K^+^ current. All values were found to be not significantly different between control and mutant *Wbp2* mice (*t*-test performed using single data points).
