# Supplementary figures and images for: Wbp2 is required for normal glutamatergic synapses in the cochlea and is crucial for hearing
Source: EMBO Mol Med. 2016 Feb 8;8(3):191–207. doi: 10.15252/emmm.201505523 (PMC4772953; doi:10.15252/emmm.201505523)

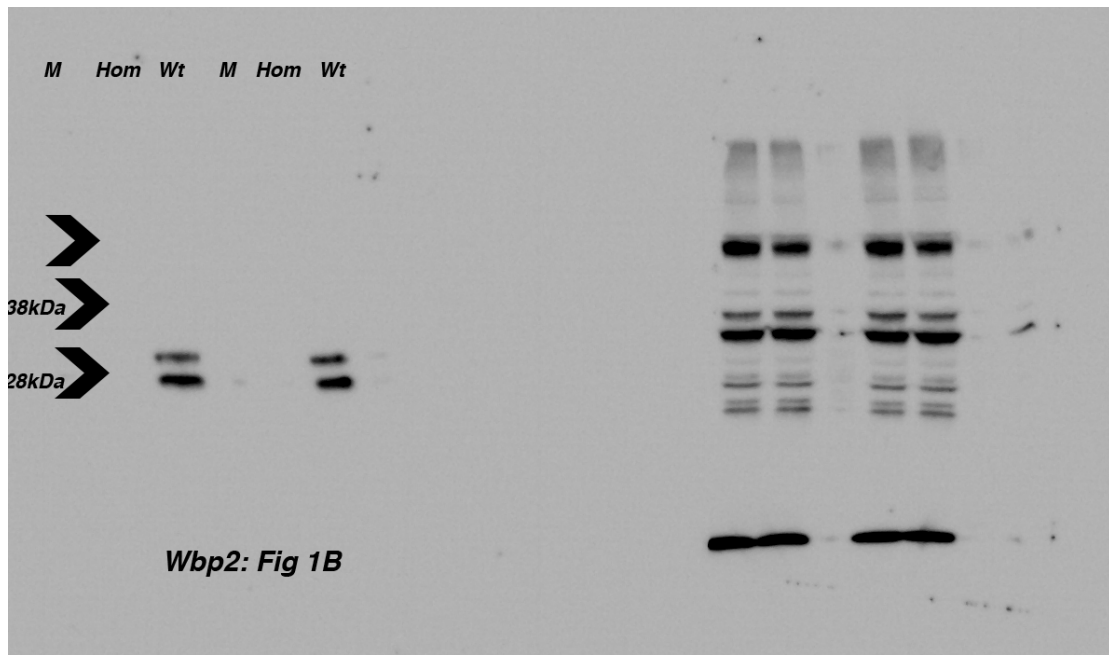

This blot is not in the paper

Fig 1B Wbp2 expression in brain

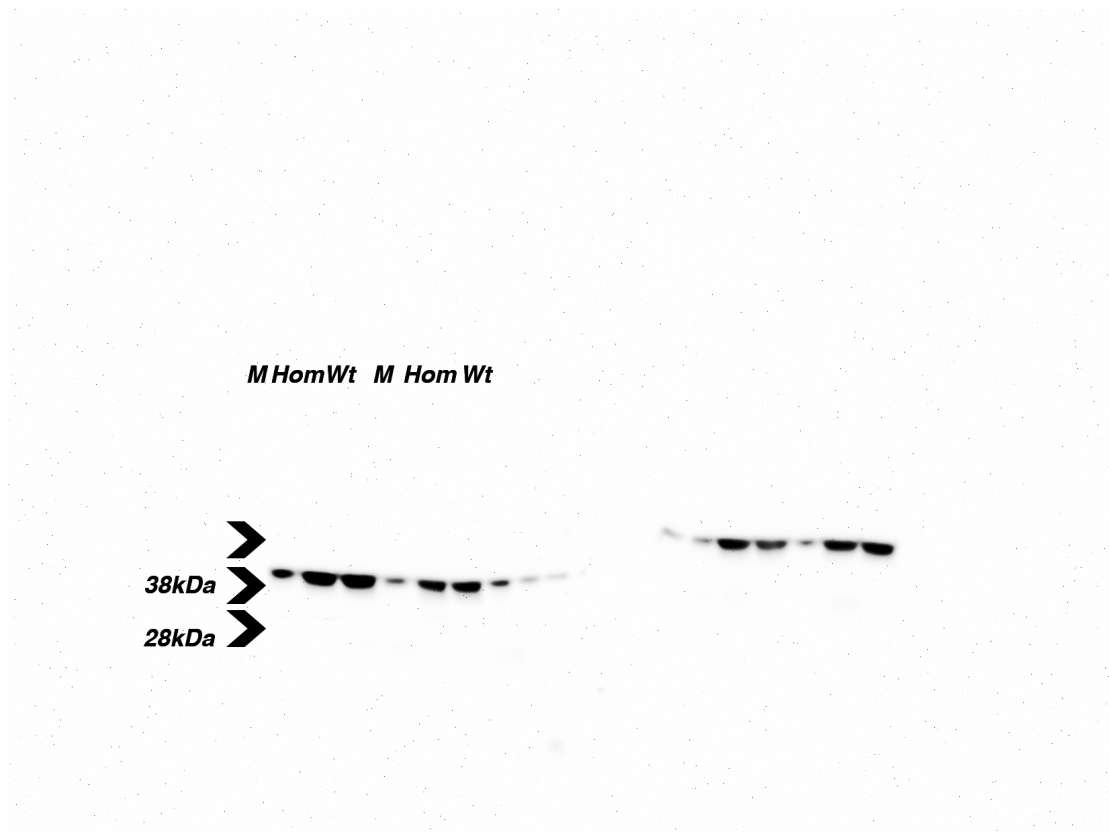

Fig 1B  $\beta$ -Tubulin

Supplement: Supplementary file 4 — Source Data for Figure 1 [file EMMM-8-191-s003.pdf]

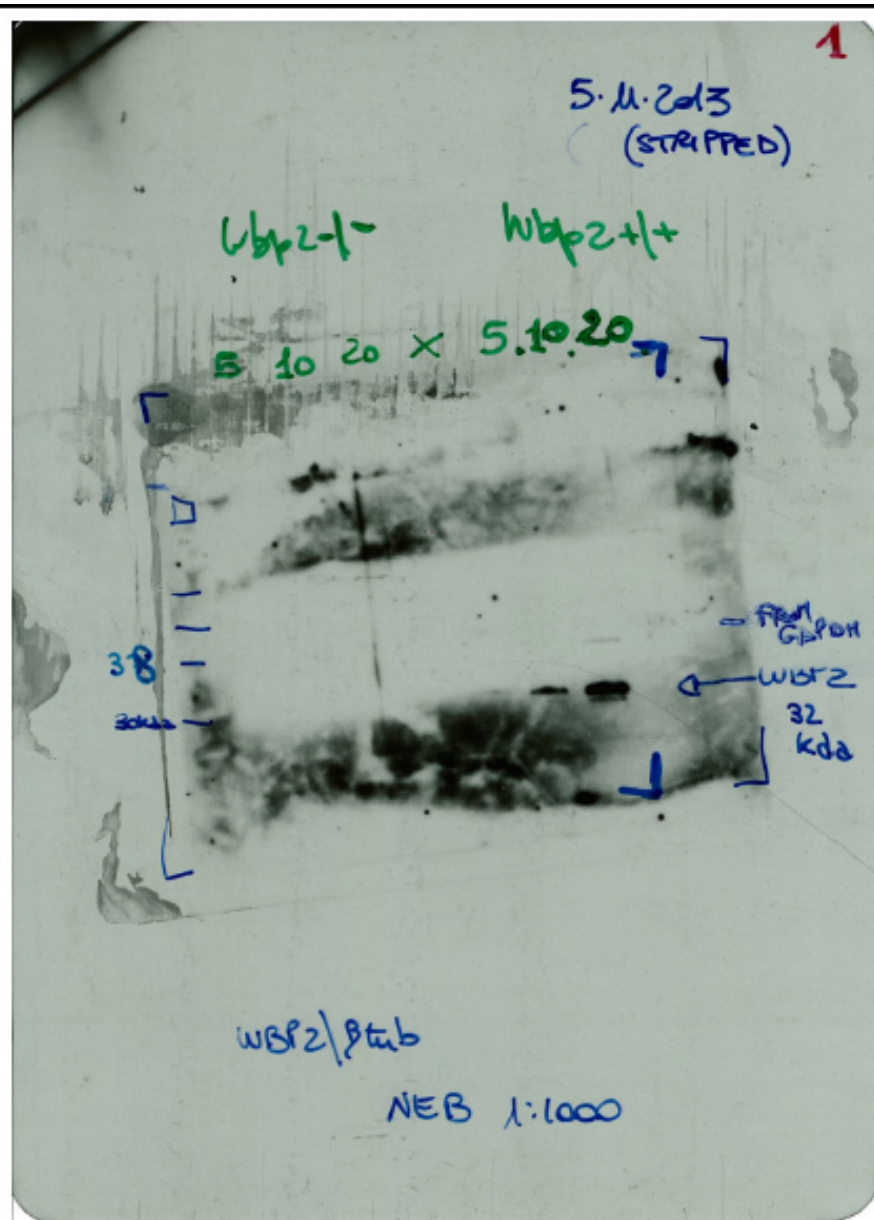

Fig 4D WBP2

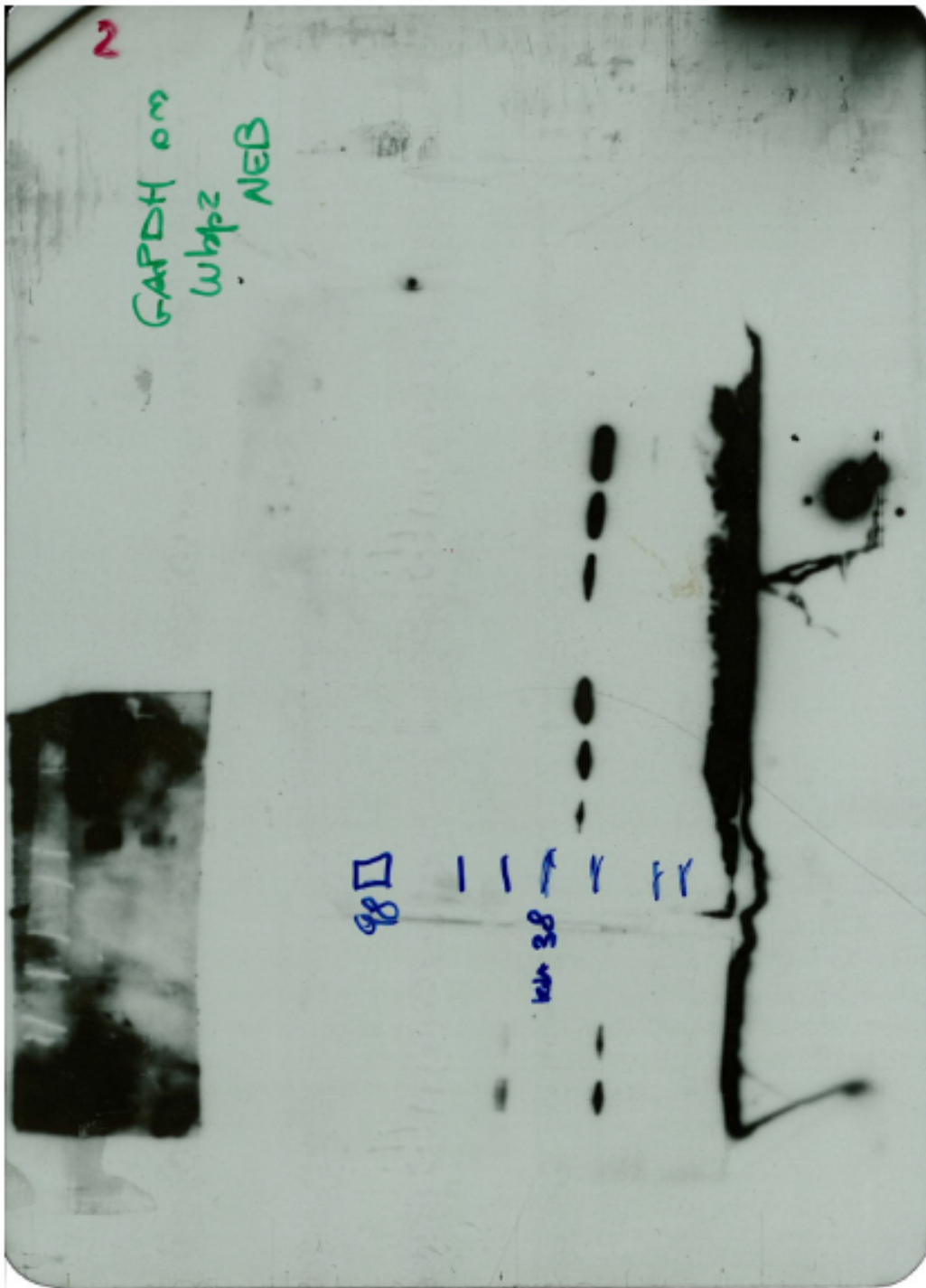

Fig 4D GAPDH

Supplement: Supplementary file 5 — Source Data for Figure 4 [file EMMM-8-191-s004.pdf]

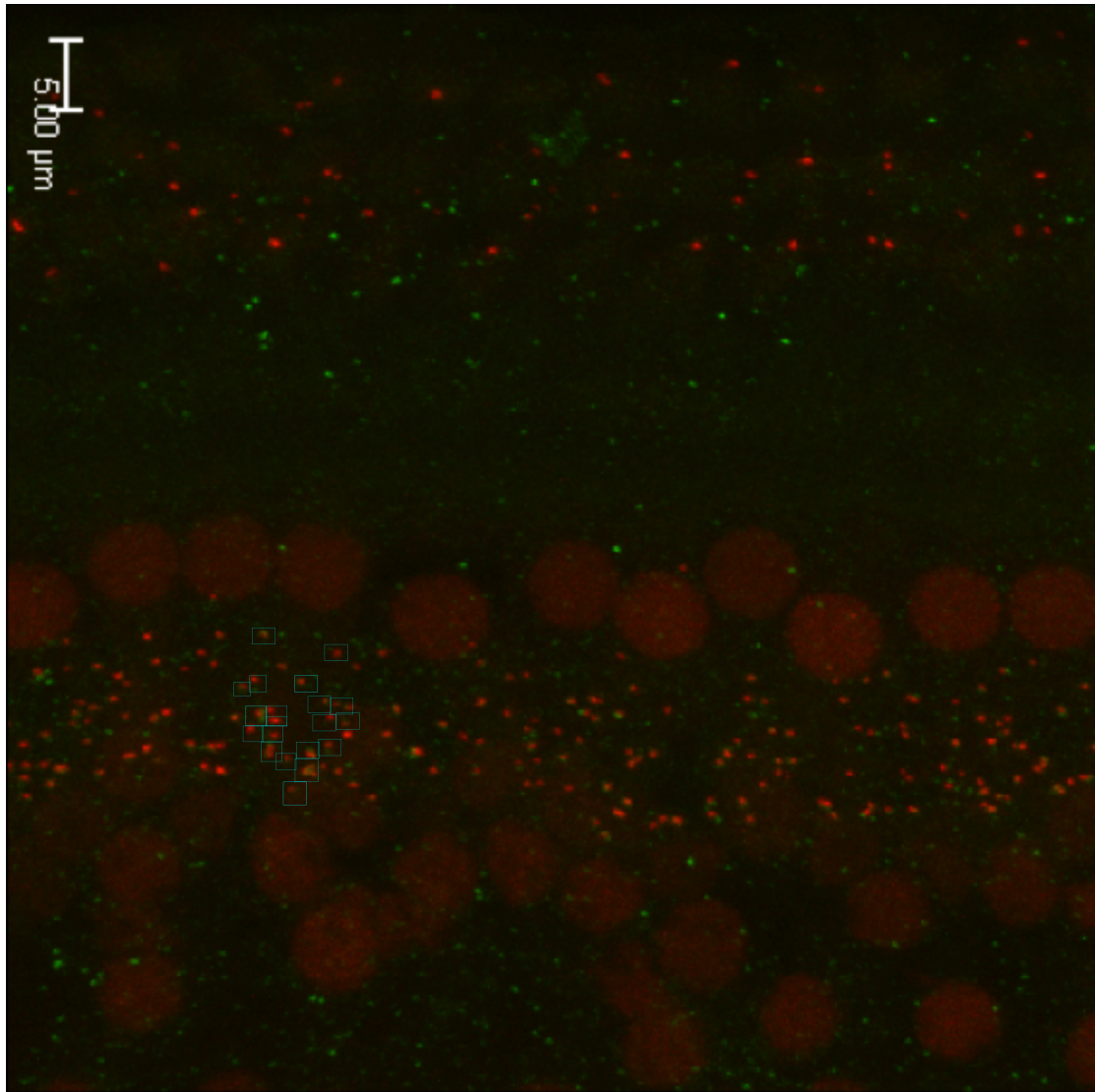

Fig6A Wt

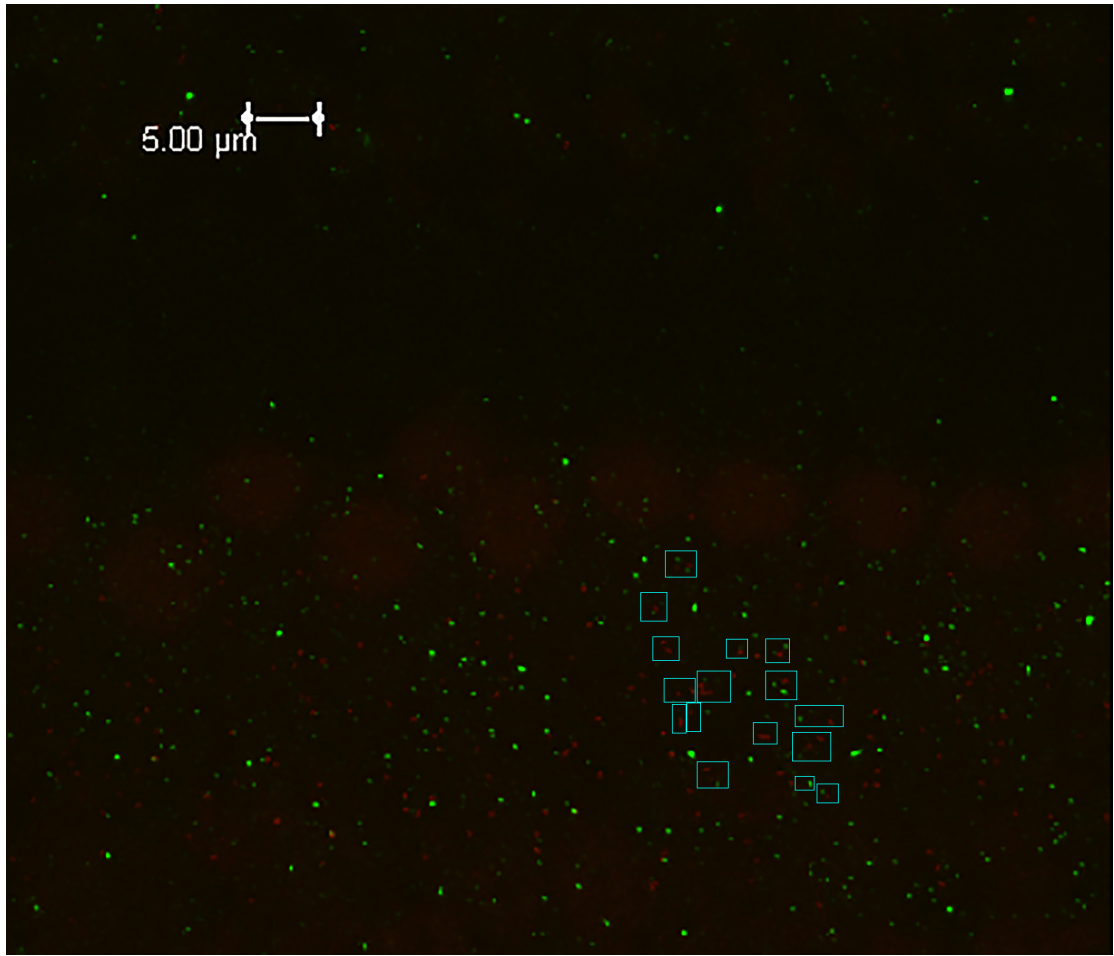

Fig6A Hom

Supplement: Supplementary file 7 — Source Data for Figure 6 [file EMMM-8-191-s006.pdf]

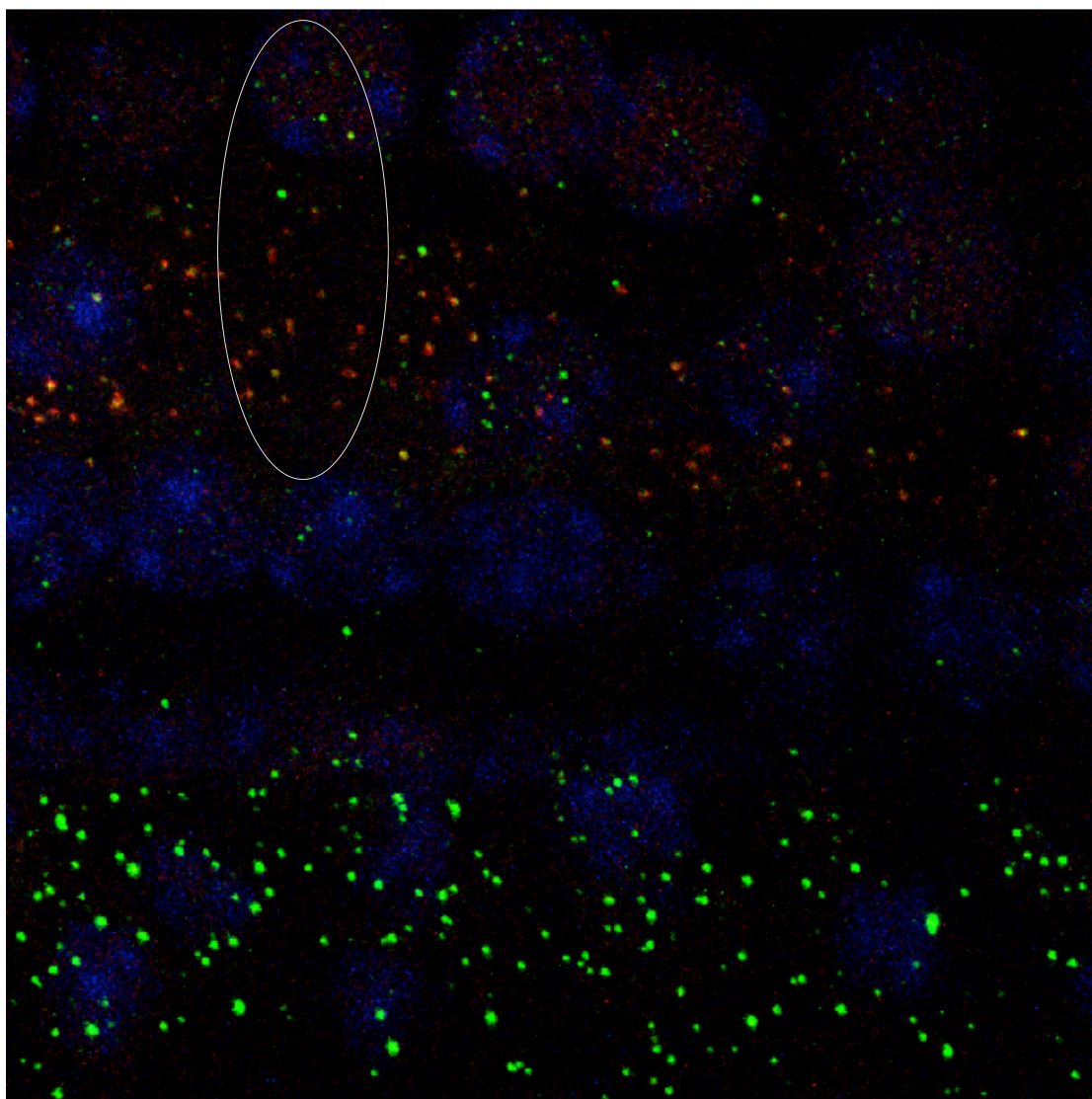

Fig 7C Wt

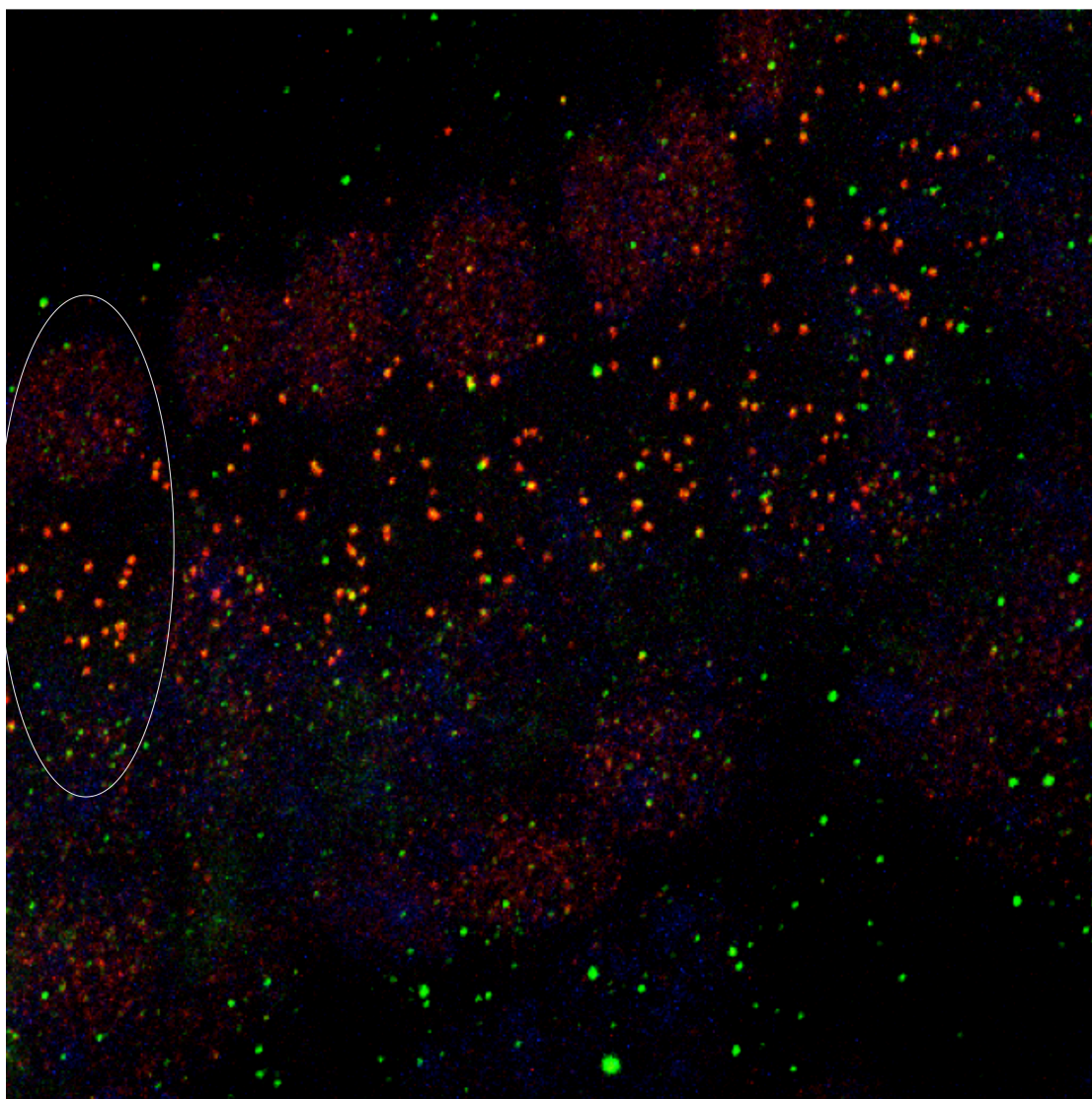

Fig7C Hom

Supplement: Supplementary file 8 — Source Data for Figure 7 [file EMMM-8-191-s007.pdf]
