# Supplementary material for: Wbp2 is required for normal glutamatergic synapses in the cochlea and is crucial for hearing
Source: EMBO Mol Med. 2016 Feb 8;8(3):191–207. doi: 10.15252/emmm.201505523 (PMC4772953; doi:10.15252/emmm.201505523)
Supplement: Supplementary file 6 — Source Data for Figure 5 [file EMMM-8-191-s005.pdf]

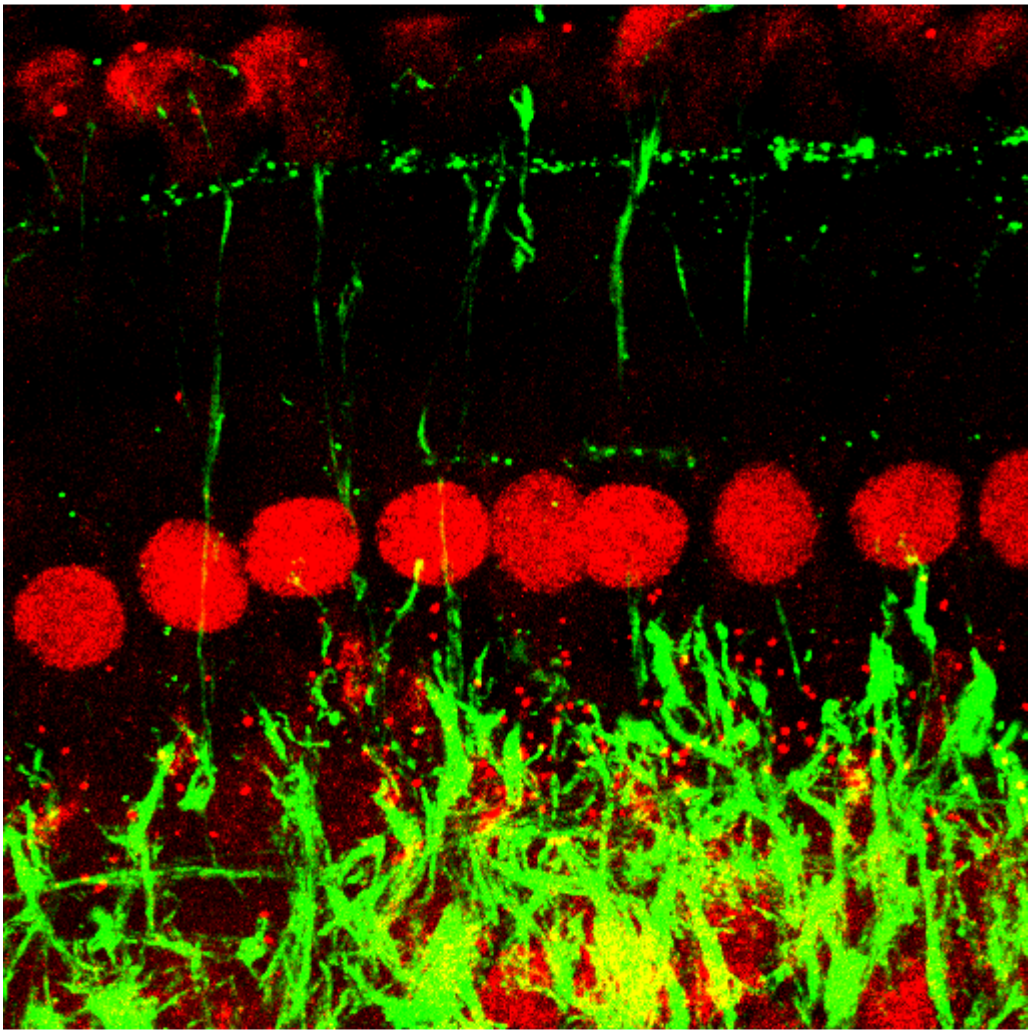

Figure 5A, panel 1

Wt, 24kHz

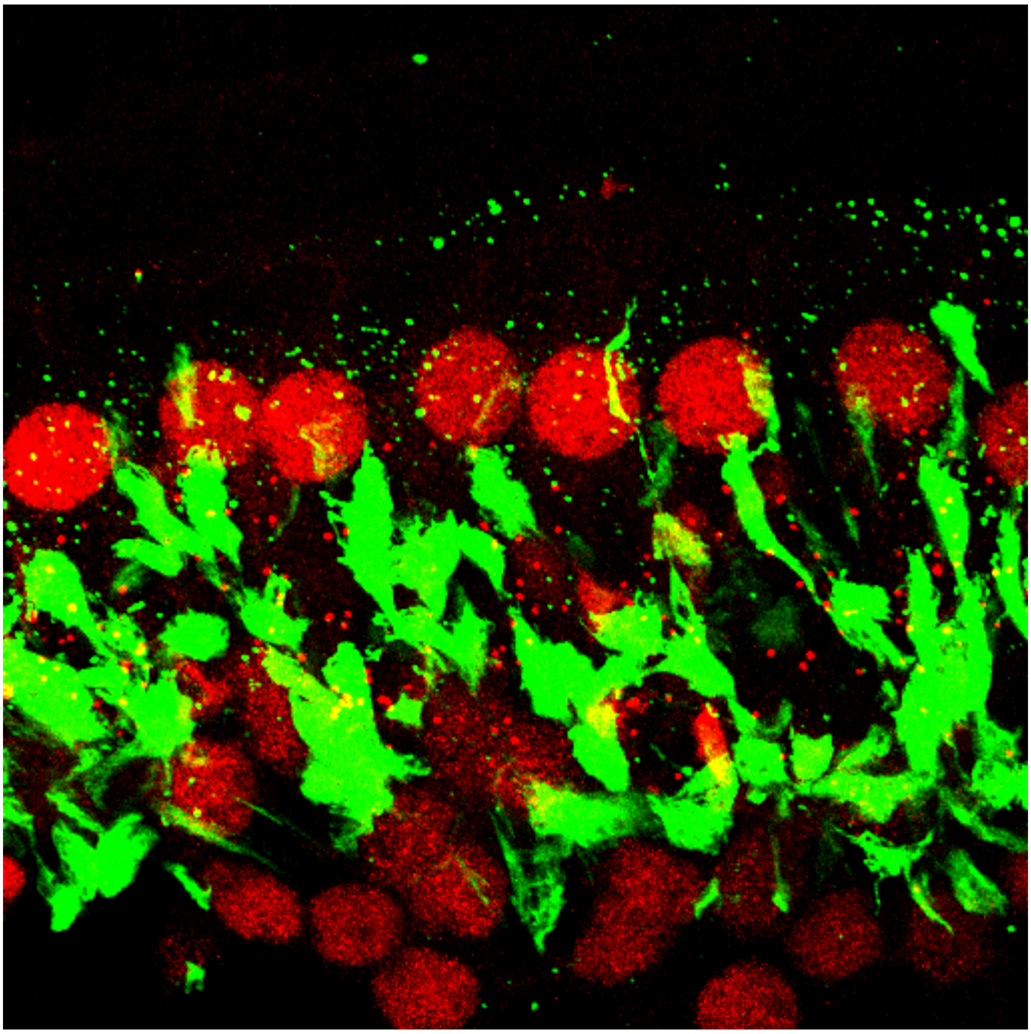

Figure 5A, panel 2

Hom, 24kHz

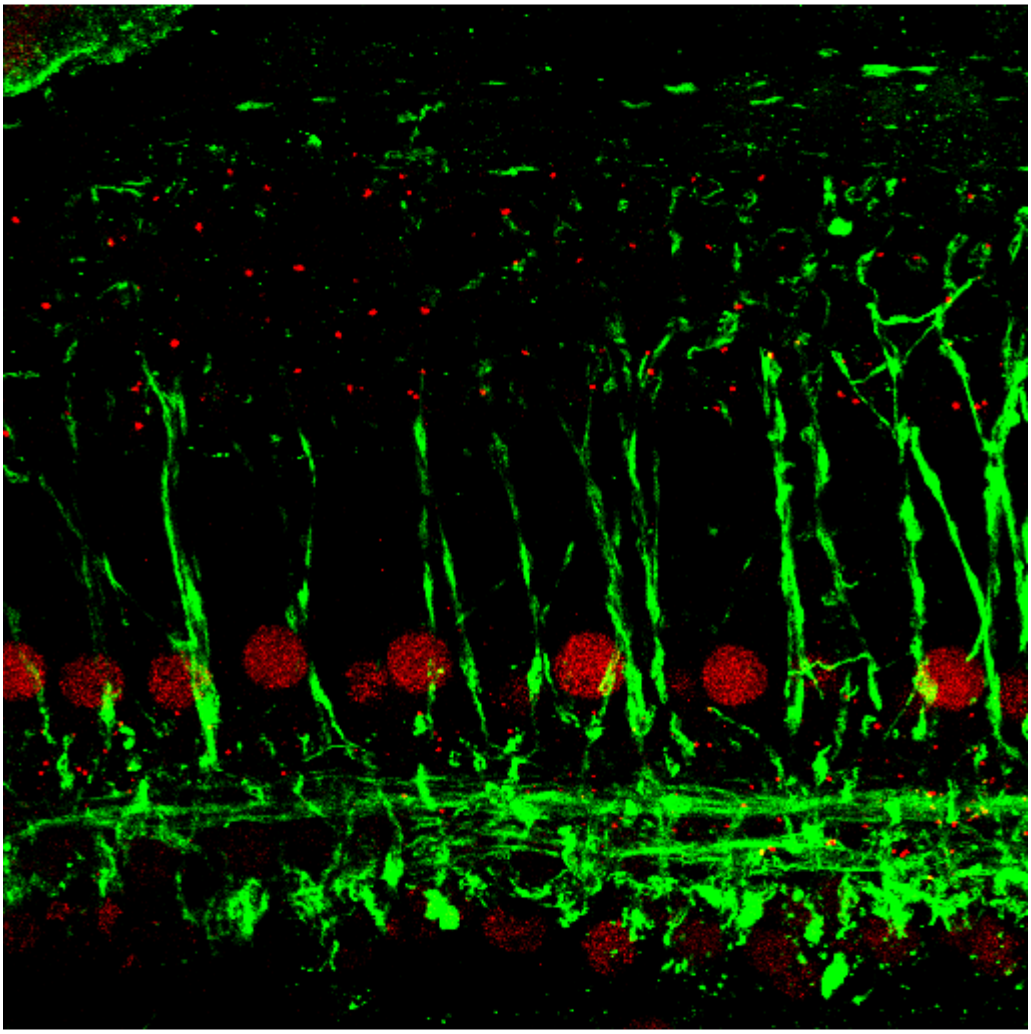

Figure 5A, panel 3

Wt, 24kHz

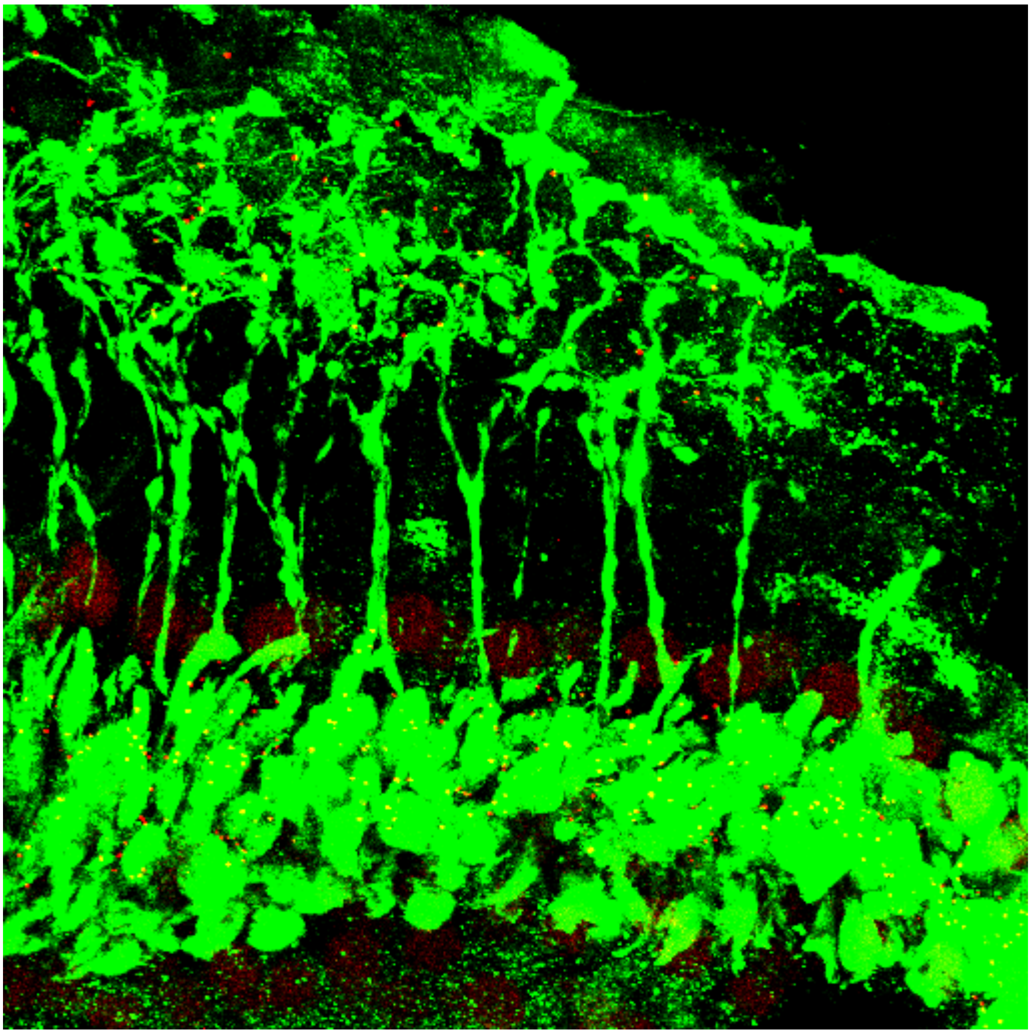

Figure5A, panel4

Hom, 24kHz

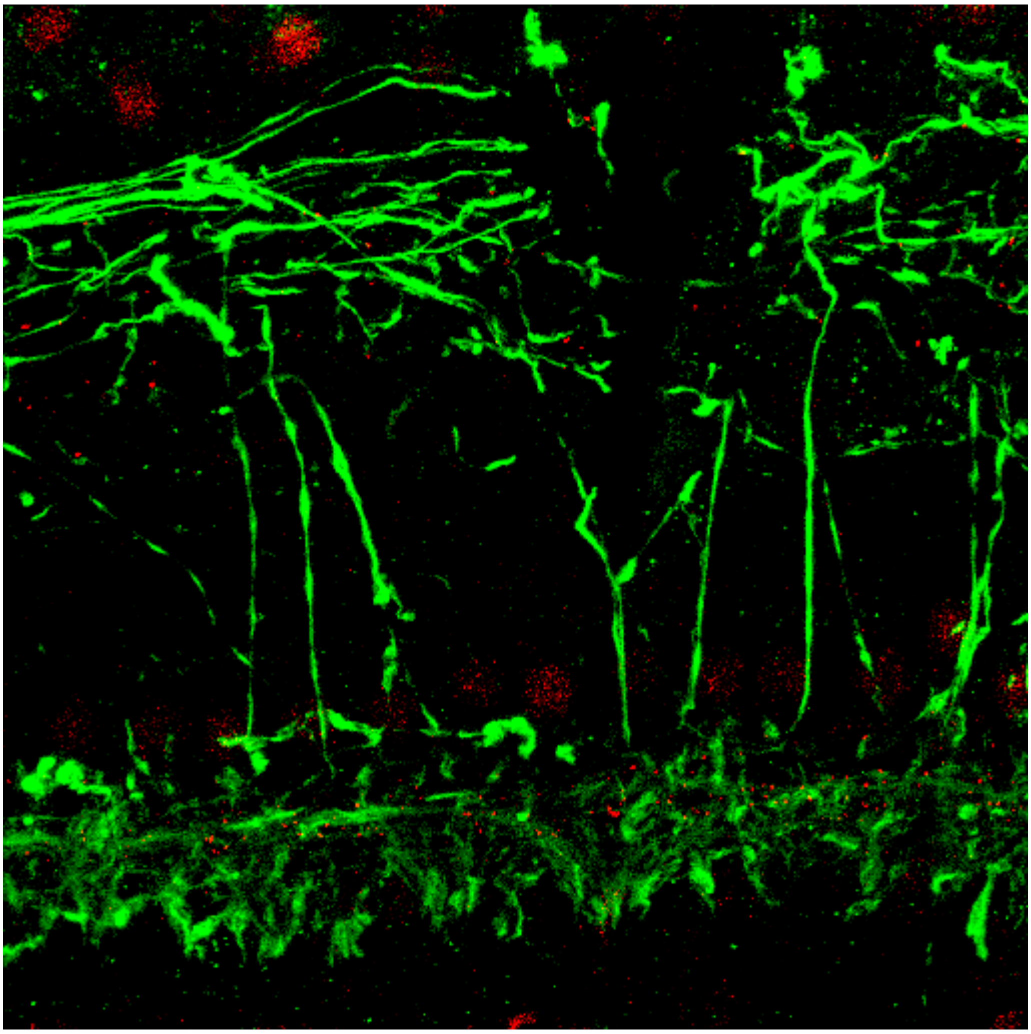

Figure 5A, panel 5

Wt, 9kHz

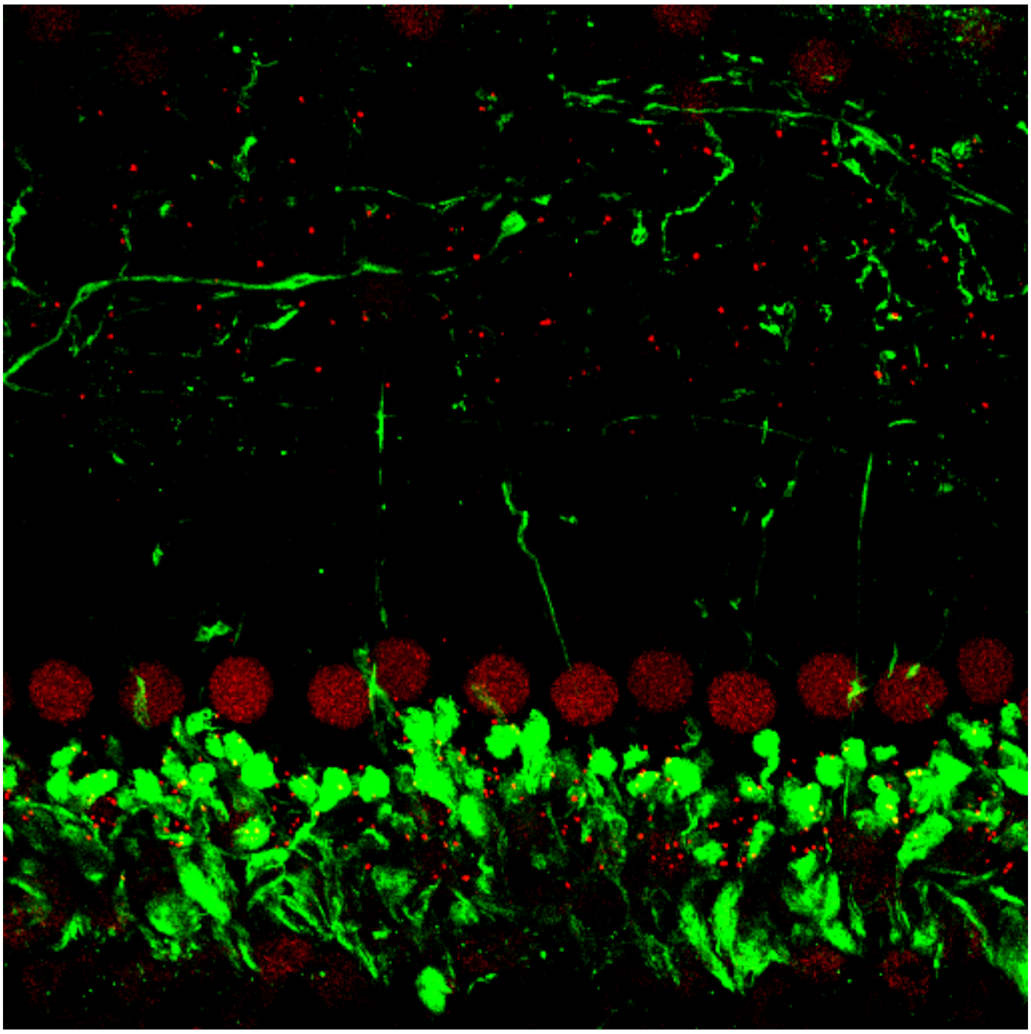

Figure 5A, panel 6

Hom, 24 kHz

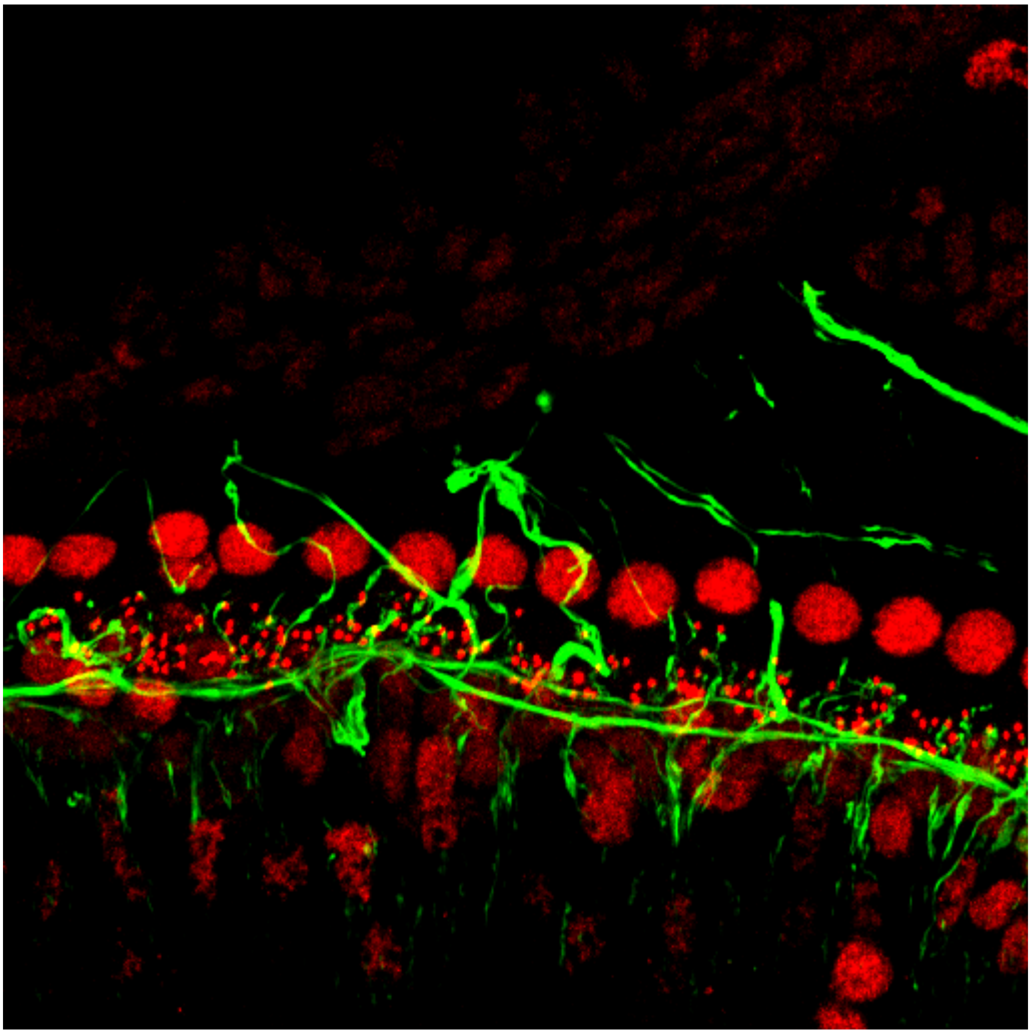

Figure5D, panel 1

Wt, 9kHz

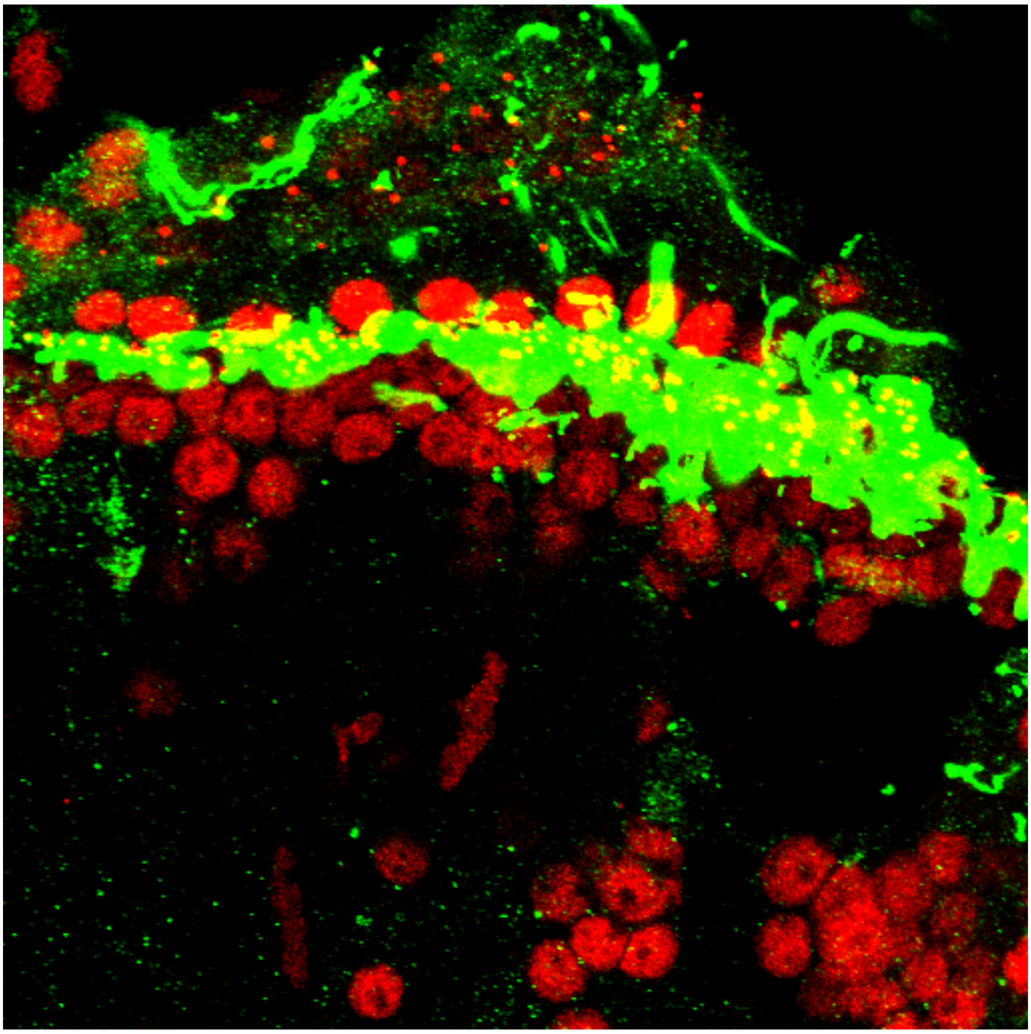

Figure5D, panel 2

Hom, 9kHz

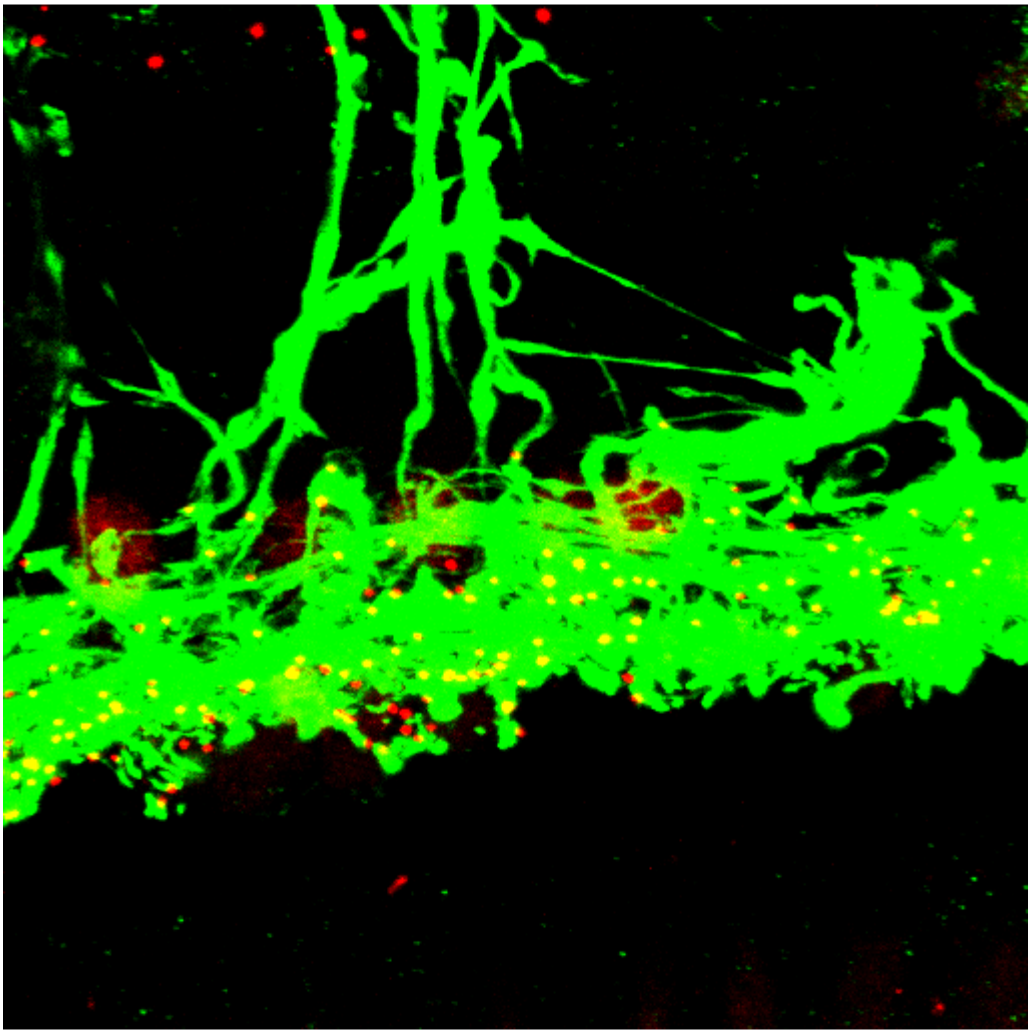

Figure 5D, panel 3

Wt, 24 kHz

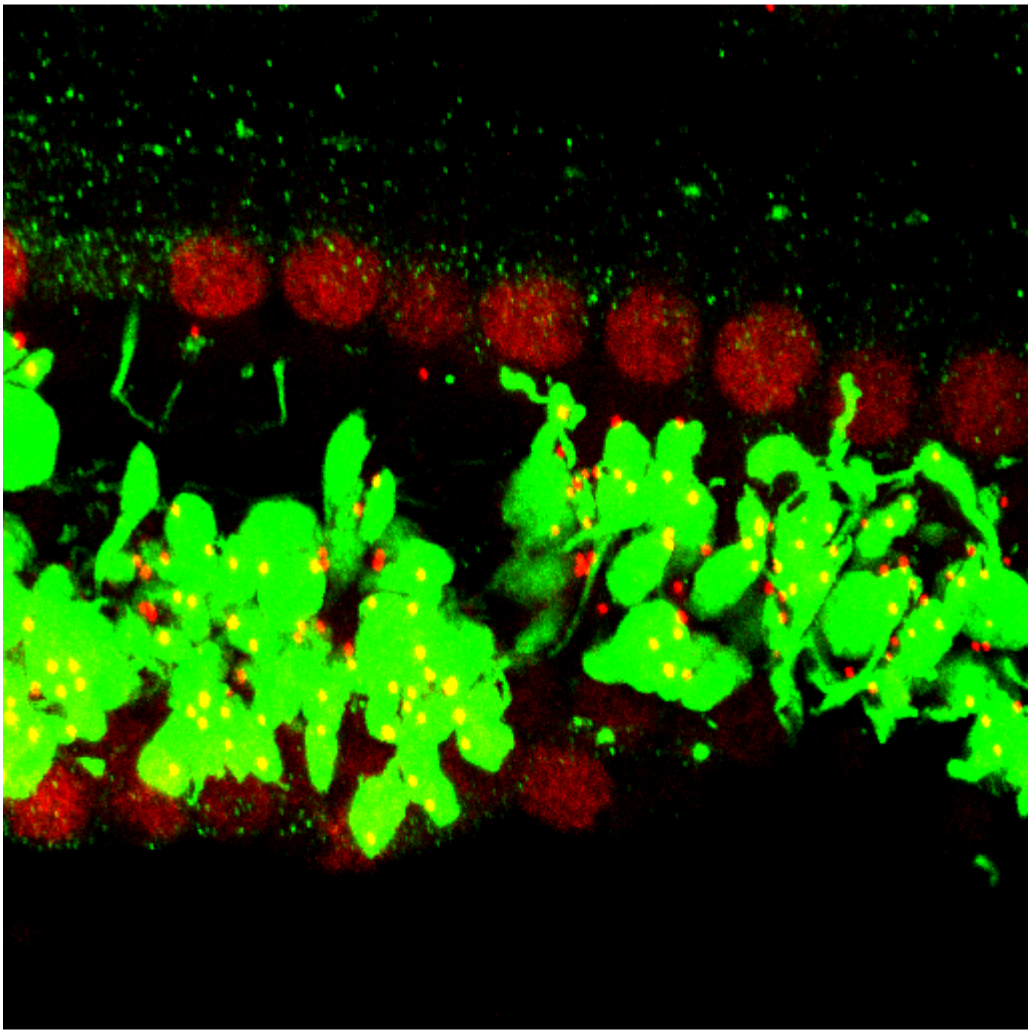

Figure5D, panel 4

Hom,24 Khz
